# A prospective cross-sectional study assessing teaching of interventional radiology across 20 Australian medical schools, endorsed by the Australian Medical Students Association

**DOI:** 10.1186/s42155-022-00344-9

**Published:** 2022-12-20

**Authors:** Warren Clements, Adil Zia, Abhishekh Srinivas, Jasmine Davis, Gerard S. Goh

**Affiliations:** 1 Department of Radiology, Alfred Health, 55 Commercial Rd, Melbourne, VIC 3004 Australia; 2 Department of Surgery, Monash University Central Clinical School, Melbourne, Australia; 3 National Trauma Research Institute, Melbourne, Australia; 4 Australian Medical Students Association, Australian Capital Territory, Barton, Australia

**Keywords:** Student, University, IR, Interventional radiology

## Abstract

**Background:**

Existing literature from around the world has shown that teaching of Interventional Radiology (IR) to medical students remains suboptimal. Despite calls for improvement at a “grass-roots” level, most IRs find that junior doctors have limited or no knowledge of IR, and thus reduced awareness of potential IR treatments for their patients or contemplating IR as a future career. The aim of this study was to survey current medical students to assess perception of whether a wider variety of medical schools are integrating IR into their curriculum, from universities all across Australia.

This was a prospective cross-sectional study of members of the Australian Medical Students Association (AMSA) from across Australia. Students were given a 14-question survey of current university teaching and students’ knowledge of the discipline of IR. The primary outcome was perception of current teaching and knowledge of IR. Secondary outcomes include awareness of technical, clinical, and other duties of IRs.

**Results:**

Surveys were sent in a newsletter and posted on the AMSA Facebook page to their members. 82 responses were received via students from 20 out of 23 Australian medical schools. 61% of students described poor or no knowledge of IR. Teaching of IR was significantly worse than diagnostic radiology (*p* < 0.001), only 12% suggested that current IR teaching was adequate, and 99% suggested that IR teaching could be improved. Only 11% of students would consider a career in IR.

**Conclusions:**

Medical student perception of exposure to IR is poor compared to diagnostic radiology. Better awareness may lead to improved referral patterns for patients and more career interest in IR.

**Supplementary Information:**

The online version contains supplementary material available at 10.1186/s42155-022-00344-9.

## Introduction

Interventional Radiology (IR) has evolved from the radiology specialty (Clements et al. 2020 Jun), offering therapeutic options for patients with equivalent efficacy to surgical alternatives, typically with less invasiveness, shorter recovery, and lower cost than for traditional open surgery (Chong et al. 2022 Apr). On some occasions, IR also offers primary care treatments with no surgical alternative (Clements et al. 2020 Jun; Chong et al. 2022 Apr).

Radiology trainees rotate through IR as a part of their broader training requirements, and those seeking to pursue a career in IR undertake dedicated post-fellowship training (Clements et al. 2020 Jun). This includes not just performing advanced image-guided interventions, but also broader clinical skills including peri-procedural management, ward management, and outpatient assessment (Clements et al. 2020 Jun). Recent growth in IR has allowed more patients to access these procedures (Clements 2022 Mar), necessitating the need for a larger IR workforce and greater awareness of the benefits of IR procedures.

Current medical students are tomorrow’s medical colleagues. They are future specialists that will work in a multidisciplinary team for patients, and future referrers of patients who may benefit from interventional treatment (Lee and Lee 2018 Feb). For these students to know how and when to refer, it is important they understand the breadth of medical specialties, the roles of these specialties, and their strengths and limitations. From an IR perspective, if patients are to benefit from minimally-invasive procedures, then all doctors including future doctors must know about IR and its scope.

To ensure optimal clinical utilisation of imaging-guided, minimally invasive procedures, the IR specialty must continue to grow and attract high quality trainees. This is only possible if IR is well-known to medical students. It has been shown that many people decide on their specialty preference at either the medical school or a junior doctor level (Clements et al. 2019 Jan; Ghatan et al. 2010). However, literature from around the world has shown that teaching of IR to medical students remains suboptimal (Ghatan et al. 2010; O’Malley and Atherya 2012; Hoffmann et al. 2018 Jan 1; Atiiga et al. 2017; Garg 2019; Xu et al. 2021; Agrawal et al. 2019; Foo et al. 2018 Dec). In Australia, Foo et al. showed in their 2018 survey of students from 2 Victorian medical schools, that only 7% of students reported adequate teaching of IR and 59% believed that IR should be in the university curriculum (Foo et al. 2018 Dec).

In spite of recent international advocacy for improvement in early exposure of IR (Alsafi et al. 2017 Jul), most IRs anecdotally find that interns and junior doctors have limited or no knowledge of IR, and thus reduced possibility of considering IR treatments for their patients or contemplating IR as a future career. Eminent IR organisations such as the Cardiovascular and Interventional Radiology Society of Europe (CIRSE) have even developed specific curricula for medical student training (Cardiovascular and Interventional Radiology Society of Europe (CIRSE): Interventional Radiology Curriculum for Medical Students [internet]).

The aim of this study was to survey current medical students to assess the awareness, understanding and exposure of IR to medical students during their training. We aimed to focus on a broad assessment of medical students from universities across Australia.

## Methods

### Institutional review board

Approval was obtained for this prospective survey from the Alfred Hospital Human Research and Ethics Committee, number 107/19. This cross-sectional study was performed according to the STROBE guideline. The survey included a statement with implied consent given from those who chose to participate.

### Survey

A 14-question survey was prepared in an electronic format via a clickable link on the Research Electronic Data Capture (REDCap) platform (Appendix 1). The survey included age, gender, medical school, years in medical school, as well as questions about knowledge, understanding, and teaching of IR.

### Identification and contact of participants

Medical students were contacted by the Australian Medical Students Association (AMSA) who are the peak representative body for all Australian medical students (Brien et al. 2021 Oct). AMSA included the survey invitation and a link in their newsletter, and placed on a post on their Facebook webpage.

### Statistics

Data were anonymously collated in REDCap, exported into Microsoft Excel (Microsoft, USA), and presented as summary statistics or in graphical form. Difference of paired samples were compared for symmetry with McNemar’s test using SPSS Statistics (version 27.0, SPSS Inc., Chicago, Ill., USA). Independence of data groups were assessed using the Chi-Squared test using the Real Statistics Resource Pack software for Excel 365. A two-tailed *p*-value of less than 0.05 was considered significant.

## Results

### Summary statistics

A total of 82 students responded to the survey from 20 Australian medical schools (Table 1). Summary statistics are shown in Table 2. The median year level of respondents was 4^th^ year (IQR 2), and the year levels were left-skewed with 94% coming from years 3–5 which are generally the clinical years and only 6% coming from the pre-clinical years.

**Table 1:** Universities where responding students are currently enrolled

Name of university
Bond University
Curtin University
Deakin University
Flinders University
Griffith University
James Cook University
Monash University
University of Adelaide
University of Melbourne
University of Newcastle
University of New England
University of New South Wales
University of Notre Dame (Sydney)
University of Queensland
University of Sydney
University of Tasmania
University of Western Australia
University of Western Sydney
University of Wollongong
Western Sydney University

**Table 2:** Summary statistics of respondents

Statistic	Value
Total respondents	82
Year level (median, IQR)	4 (2)
Gender (number, percentage)	Female: 45 (55%)
	Male: 36 (44%)
	Other: 1 (1%)
Undergraduate (number, percentage)	45 (55%)
Age range (mode)	21–25 years
Urban location (number, percentage)	70 (85%)
Students that would currently consider a career in Interventional Radiology based on their current knowledge (number, percentage)	9 (11%)

The majority of respondents were female (55%), from an undergraduate training pathway (55%), and in an urban clinical school (85%). The mode of the age range was 21–25 years.

Only 9 students (11%) would currently consider a career in IR based on their existing knowledge. Table 3 shows the general demographic of these 9 students interested in IR. There was a trend to smaller proportion of female students (general cohort 55%, interest in IR 44%, *p* = 0.477), and a trend to a smaller proportion from undergraduate training (general cohort 55%, interest in IR 33%, *p* = 0.168). The year level, age, and region of clinical school were similar to the general population.

**Table 3:** Summary statistics of students that would currently consider a career in Interventional Radiology

Statistic	Value
Total respondents (number, percentage of total)	9 (11%)
Year level (median, IQR)	4 (2)
Gender (number, percentage)	Female: 4 (44%)
	Male: 5 (56%)
	Other: 0 (0%)
Undergraduate (number, percentage)	3 (33%)
Age range (mode)	21–25 years
Urban location (number, percentage)	8 (89%)

### Understanding of IR

**Fig. 1:**
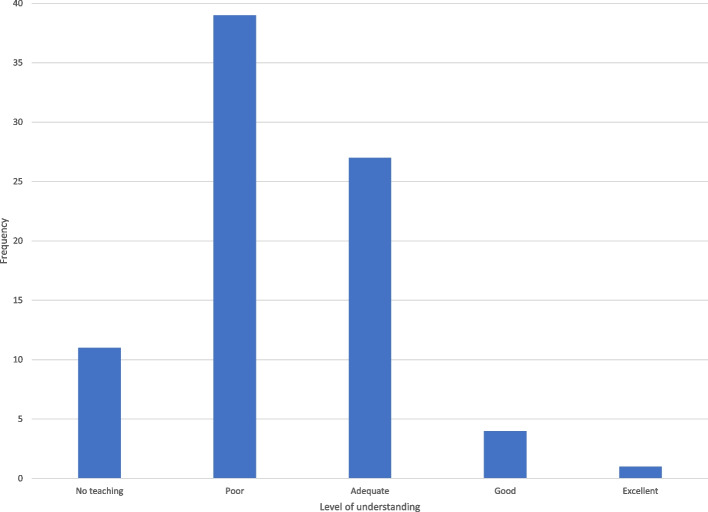
Bar graph showing the current understanding of interventional radiology from respondents

Figure 1 shows the current understanding of IR. The median response was “poor” and the bar graph is right-skewed, with 72 respondents (88%) describing no teaching or poor understanding of IR, and only 10 students (12%) describing IR teaching as “adequate”.

### Teaching of IR

**Fig. 2:**
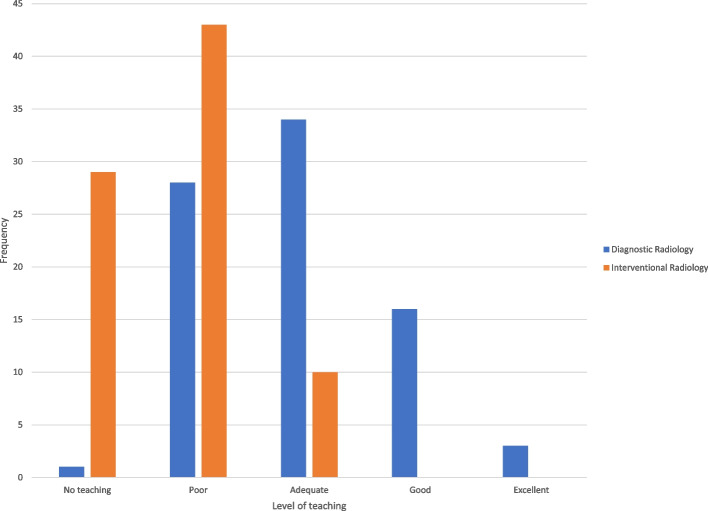
Bar graph comparing the level of teaching of interventional radiology compared to diagnostic radiology

Figure 2 compares the teaching of IR with the teaching of general diagnostic radiology (DR). Diagnostic radiology teaching has an approximately normal distribution and only 29 (35%) report no or poor teaching of diagnostic radiology. However, the teaching of IR was right skewed with 72 (88%) reporting either no or poor teaching. The difference in the symmetry of the IR and DR graphs comparing “no or poor teaching” with “adequate teaching or above” was significant, *p* < 0.001.

### Clinical role of IR

**Fig. 3:**
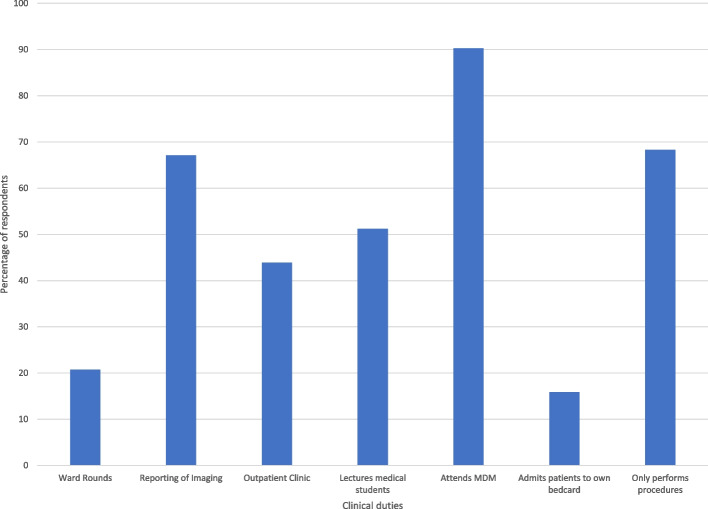
Bar graph showing the current understanding of different interventional radiology clinical duties

Figure 3 shows responses regarding different clinical duties that students considered an IR may be involved in. 90% of respondents correctly assumed that IRs attend multidisciplinary meetings, 67% correctly identified that IRs report diagnostic imaging, and 51% correctly suggested that IRs lecture medical students. However, 68% incorrectly identified that IRs only perform procedures by referral from other specialists. The understanding of IRs attending outpatient clinic (44%), ward rounds (21%) and admitting patients to their own bed card (16%) were all poor.

### IR procedures

**Fig. 4:**
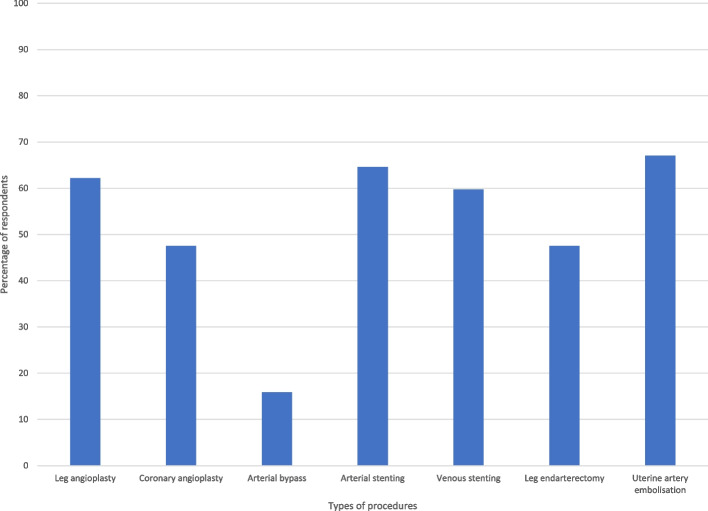
Bar graph showing the current understanding of different procedures, some of which are performed by interventional radiologists

Figure 4 shows different types of procedures. The majority of students correctly identified that IRs perform leg angioplasty (62%), arterial stenting (65%), venous stenting (60%) and uterine artery embolisation (67%). However, students also incorrectly identified that IRs perform coronary angioplasty (48%), arterial bypass (16%) and endarterectomy (48%).

### Need for improvement

**Fig. 5:**
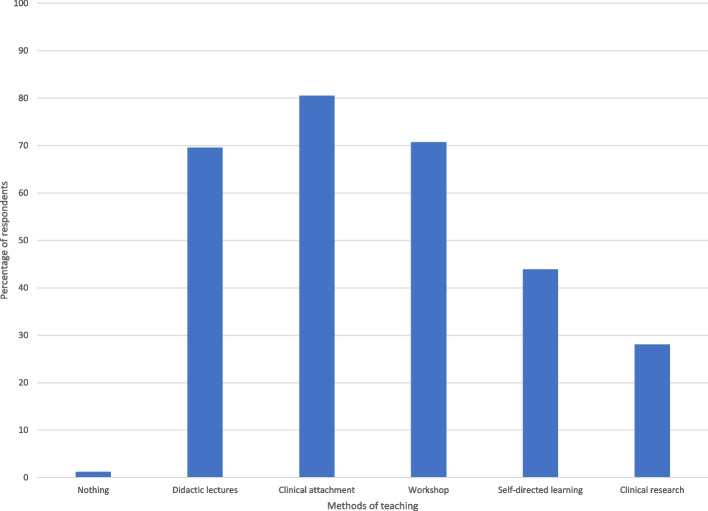
Bar graph showing ways students suggest interventional radiology teaching could be improved in medical schools

Figure 5 shows ways students suggest that IR teaching and education could be improved. A suggestion for improvement was given by 99% of respondents with only 1 student suggesting nothing could be improved. The same student also selected that they had “no understanding of IR” received “no teaching of IR” and did not correctly answer any of the duties that IRs perform other than procedures.

## Discussion

This study gives a subjective indication of medical students' experiences and awareness of IR through their exposure to teaching, including students from 20 out of 23 different medical schools, and from all Australian states.

The results show that 50 students (61%) considered their level of understanding of IR to be “none” or “poor” (Fig. 1), noting that students will inherently compare this to their level of understanding of other specialties that they are familiar with. As a survey on student perception, the results suggest that IR understanding is far lower than what the authors would consider adequate and correlates with international literature (Ghatan et al. 2010; O’Malley and Atherya 2012; Hoffmann et al. 2018 Jan 1; Atiiga et al. 2017; Garg 2019; Xu et al. 2021; Agrawal et al. 2019; Foo et al. 2018 Dec). This is concordant with similar results in Fig. 2, where 72 students (88%) considered their perception of IR teaching to be “none” or “poor”, and this perception of teaching was significantly lower than the perception of teaching of diagnostic radiology (*p* < 0.001). 99% of students suggested IR teaching and education could be improved. It can be assumed that students are attending relevant teaching opportunities given their willingness to respond to the survey, and thus we believe that the results imply a relative deficiency in teaching of IR compared to DR, but also likely to be deficient compared to other well-known specialties.

There are likely to be a number of reasons for this disparity. First, in keeping with existing international surveys, IR remains an under-acknowledged specialty and has not made its way into mainstream medical teaching. This is likely compounded by a small number of IRs who are all busy clinicians, and it is unlikely that there is a large representation of IRs amongst clinical university trainers. As such, there is not only a lack of teaching but likely a lack of mentorship, and it is thus not surprising that only 11% of students surveyed would consider a career in IR.

What is concerning is that these results haven’t changed from previous studies including Foo et al. who suggested that only 7% of students they surveyed reporting adequate teaching of IR from 2 Victorian medical schools (Foo et al. 2018 Dec). A recent large study from Brien et al. presented the results of a wide survey of medical students from 11 countries across Europe. They showed that that only 10% of students had heard of IR, and two-thirds of students had no formal exposure to interventional radiology (Brien et al. 2021 Oct). What is most worrying is that there has also been no change to this trend dating back over 10 years from surveys across the world (Ghatan et al. 2010; O’Malley and Atherya 2012; Hoffmann et al. 2018 Jan 1; Atiiga et al. 2017; Garg 2019; Xu et al. 2021; Agrawal et al. 2019; Foo et al. 2018 Dec; Brien et al. 2021 Oct).

In assessing the technical side of IR, students did have knowledge of different procedures that IR does (including angioplasty, stenting, and embolisation). However, students also did not know that cardiac and open surgical procedures were not performed by IRs. This suggests either an acquiescence bias to the responses, and/or just a generally low understanding of what an IR can offer patients, similar to what has been reported in prior studies from the European Trainee Forum (ETF) (Brien et al. 2021 Oct). This further supports the low understanding of the duties of IR even though students at a median of 4th year would likely have had some exposure to surgical and cardiac procedures given they are relatively mainstream compared to IR procedures.

In assessing the clinical side of IR, students have a low understanding that IRs perform clinical duties including ward rounds, outpatient clinics, and admitting patients to their own bed card. They also incorrectly identified that IRs only perform procedures from other specialists, when in reality many general practitioners refer patients, and the range of care being offered by IRs extends beyond just procedures. This further supports a general lack of understanding of the specialty of IR including its scope and daily workflow.

In looking at student-reported ways to improve existing IR education, of the 99% of students who suggested ways to improve, they advised that a broad range of different potential measures could be employed including didactic, self-directed, clinical, and research learning techniques. This is similar to how different topics are integrated into a well-rounded and multi-modality teaching style, and these are all techniques that IR needs to harness. The ETF is a subcommittee of CIRSE which provides governance and advocacy for IR trainees including medical students and doctors in training. In their 2020 report on the status of IR training in Europe, they highlight the vital importance of teaching of IR in university faculties (Makris and (Editor). xxxx).

In supporting this forward-thinking approach, CIRSE has released a medical student curriculum (Cardiovascular and Interventional Radiological Society of Europe: Student Programme – Be InspIRed [internet]) which outlines an evidence-based document based on the scope of expert consensus, providing the roadmap for medical schools who wish to begin IR integration. CIRSE also advocates for IR training in further ways including the ETF, online training and advocacy, and dedicated webinar series (Cardiovascular and Interventional Society of Europe. ETF webinars [internet]. Accessed 30/11/22. Available from URL: https://www.cirse.org/trainees/etf-webinars/16. Australian Medical Students Association (AMSA) 2020). In addition, IRs including their college (Royal Australian and New Zealand College of Radiologists or RANZCR) and specialty societies (Interventional Radiology Society of Australasia or IRSA) should provide initiatives to incentivise students and promote training opportunities. This may be similar to the “Be inspIRed” program being offered by CIRSE (Cardiovascular and Interventional Radiological Society of Europe: Student Programme – Be InspIRed [internet]), or to support and work with AMSA. IRs should also actively seek involvement in training and education, including both clinical and research placement in partnership with their local universities.

At the 2021 RANZCR Annual Scientific Meeting, it was announced by the RANZCR President that the college would be applying to the Australian Medical Council with the aim of achieving separate specialty recognition for IR (RANZCR Inside News 2021; RANZCR). A successful application would mean IR and DR would be formally recognised as different specialties. Such a change may be a pivotal step in providing the foundation that IR needs for more formal medical school integration and will lead to a larger IR workforce which will allow staff to filter into universities similar to colleagues from physician and surgical specialties.

Muzumdar et al. suggests a lack of exposure is behind the lack of drive towards IR (Muzumdar et al. 2019) and as such, these initiatives are vital for patients and future doctors to understand the cost-effectiveness, short recovery times, and low complication rates that can be offered by IR. Shaikh et al. also showed that even something as simple as a short 10-h teaching curriculum can increase the knowledge and understanding of IR within medical students (Shaikh et al. 2016 Apr). The authors advocate for changes to begin with immediate effect in worldwide medical curricula, including Australia.

This study must be interpreted within its limitations. The overall response rate was low and likely to represent a number of factors. Firstly, as an online survey embedded within a newsletter there may be many students who did not read or see the survey. In addition, access to the Facebook page requires a specific account and the post may be lost within a range of other posts. There is certainly a significant response bias in that students don’t know much about IR and thus didn’t want to reply to something they didn’t fully understand, as shown in previous studies (Brien et al. 2021 Oct). Conversely, this low rate may actually reflect the survey conclusions, showing that people aren’t responding because their knowledge of IR is low and is supported by the 2020 report from CIRSE and the ETF (Makris (Editor)). Response rates may have been improved with incentives, but this introduces its own courtesy bias. In addition to the low rate, the authors also acknowledge existing studies in Australia on the topic (Clements 2022 Mar), however conversely this study presents a wider response from 20 different medical schools rather than 2, with a more national message being present in this manuscript. This study also assesses perception which is an indirect maker of measuring the curricula, and another method would be to assess medical schools themselves. We also acknowledge that the survey assessed predominantly vascular IR procedures and did not full address the scope of non-vascular IR, interventional neuroradiology, and interventional oncology procedures.

We also acknowledge that the responding medical students were predominantly from their senior years, similar to what has been reported in similar international surveys (Ghatan et al. 2010; O’Malley and Atherya 2012; Hoffmann et al. 2018 Jan 1; Atiiga et al. 2017; Garg 2019; Xu et al. 2021; Agrawal et al. 2019; Foo et al. 2018 Dec). This suggests that the exposure these students have had may not have come until their clinical time when they have made referrals to IR. Responses from junior medical students may be different, possibly even worse as the teaching is likely to be even less. There are several issues to consider with this. First, while this study assessed perception of teaching, it did not consider what type of teaching students had. There is likely to be a vast difference in the exposure students have depending on whether they are rotating to a large centre with advanced IR, a small centre with mainly basic procedures, or just receiving lectures with no practical exposure. While this is also a bias for all specialties based on the current model of rotating clinical teaching, IR teaching is arguably more vulnerable given the small workforce and large differences in work type and acuity between centres. This bias should be considered for the students who replied to the survey and their perception of IR duties (Figs. 3 and 4), but this also highlights why it is important to improve medical student teaching.

## Conclusions

This survey from students attending a broad range of medical schools across Australia suggests that medical student perception of exposure to IR is poor compared to diagnostic radiology. Existing evidence-based curricula are available, and it is time for medical schools to partner with AMSA, RANZCR, and IRSA to initiate changes. Better awareness may lead to improved referral patterns for patients and more career interest in IR. It may be that formal recognition of IR as a distinct specialty is required before this goal can be realistically achieved.

## Supplementary Information

**Additional file 1: Appendix 1.** Medical student survey questions.

## Data Availability

The datasets generated and/or analysed during the current study are not publicly available as this was not a part of the IRB approval process, please contact the corresponding author for additional information.

## Abbreviations

IR: Interventional Radiology
CIRSE: Cardiovascular and Interventional Society of Europe
AMSA: Australian Medical Students Association
RANZCR: Royal Australian and New Zealand College of Radiologists
IRSA: Interventional Radiology Society of Australasia
REDCap: Research Electronic Data Capture
STROBE: Strengthening the Reporting of Observational Studies in Epidemiology
ETF: European Trainee Forum
