# Supplementary material for: A prospective cross-sectional study assessing teaching of interventional radiology across 20 Australian medical schools, endorsed by the Australian Medical Students Association
Source: CVIR Endovasc. 2022 Dec 20;5:66. doi: 10.1186/s42155-022-00344-9 (PMC9763521; doi:10.1186/s42155-022-00344-9)
Supplement: Supplementary file 1 — Additional file 1: Appendix 1. Medical student survey questions. [file 42155_2022_344_MOESM1_ESM.docx]

Appendix 1: Medical student survey questions

**What university are you currently enrolled in?**

Australian national university (ANU)

Bond university

Curtin University

Deakin University

Flinders University

Griffith University

James Cook University

Macquarie University

Monash University

University of Adelaide

University of Melbourne

University of Newcastle

University of New England

University of New South Wales

University of Notre Dame (Fremantle)

University of Notre Dame (Sydney)

University of Queensland

University of Sydney

University of Tasmania

University of Western Australia

University of Western Sydney

University of Wollongong

Western Sydney University

University of Newcastle / University of New England JMP

Charles Sturt University/Western Sydney University JMP

**What is your current year level?**

Year 1

Year 2

Year 3

Year 4

Year 5

Year 6

**What is you gender?**

Female

Male

Non-binary

Transgender

Gender-diverse or Gender Queer

Questioning/Unsure

Prefer not to disclose

Other (space here to type)

**What was your entry path to medicine?**

Undergraduate

Graduate

**What is your age?**

17-20

21-25

26-30

31+

**What is your primary location of medical training?**

Urban

Rural

**How would you describe your general understanding of what an Interventional Radiologist is?**

No teaching

Poor

Adequate

Good

Excellent

**Please select the category which best describes your feeling of Diagnostic Radiology teaching during your medical studies (eg teaching of CT scans and x-rays), as compared to teaching of other topics**

No teaching

Poor

Adequate

Good

Excellent

**Please select the category which best describes your feeling of Interventional Radiology teaching during your medical studies, as compared to teaching of other topics**

No teaching

Poor

Adequate

Good

Excellent

**Please select the clinical duties which you think are performed by an Interventional Radiologist**

Ward rounds

Reporting of images

Outpatient clinic

Lectures medical students

Attends multidisclipinary cancer meetings (MDTs)

Admits patients under their own bedcard

Only performs procedures via referrals from other doctors

**Please select procedures which you think are performed by an Interventional Radiologist**

Leg vessel angioplasty

Coronary angioplasty

Arterial bypass

Arterial stenting

Venous stenting

Leg artery endarterectomy

Uterine artery embolization for fibroids

**Please select the things which you think are important to improve education about Interventional Radiology at your medical school**

Nothing

Didactic lectures

Attachment to Interventional Radiology clinical service

Hands-on workshops

Self-directed learning resources

Clinical research projects

**Would you consider a career in Interventional Radiology based on your current knowledge?**

Yes

No

Need more information

**What do you think of the career prospects for Interventional Radiologists?**

None

Poor

Adequate

Good

Excellent
